# Automatic Defect Detection for TFT-LCD Array Process Using Quasiconformal Kernel Support Vector Data Description

**DOI:** 10.3390/ijms12095762

**Published:** 2011-09-09

**Authors:** Yi-Hung Liu, Yan-Jen Chen

**Affiliations:** 1 Department of Mechanical Engineering, Chung Yuan Christian University, Chungli 320, Taiwan; 2 Photo Engineering Department, AU Optronics (AUO) Corporation, Taoyuan 325, Taiwan; E-Mail: brainchen@auo.com

**Keywords:** thin film transistor liquid crystal display, array process, defect detection, machine learning, support vector data description

## Abstract

Defect detection has been considered an efficient way to increase the yield rate of panels in thin film transistor liquid crystal display (TFT-LCD) manufacturing. In this study we focus on the array process since it is the first and key process in TFT-LCD manufacturing. Various defects occur in the array process, and some of them could cause great damage to the LCD panels. Thus, how to design a method that can robustly detect defects from the images captured from the surface of LCD panels has become crucial. Previously, support vector data description (SVDD) has been successfully applied to LCD defect detection. However, its generalization performance is limited. In this paper, we propose a novel one-class machine learning method, called quasiconformal kernel SVDD (QK-SVDD) to address this issue. The QK-SVDD can significantly improve generalization performance of the traditional SVDD by introducing the quasiconformal transformation into a predefined kernel. Experimental results, carried out on real LCD images provided by an LCD manufacturer in Taiwan, indicate that the proposed QK-SVDD not only obtains a high defect detection rate of 96%, but also greatly improves generalization performance of SVDD. The improvement has shown to be over 30%. In addition, results also show that the QK-SVDD defect detector is able to accomplish the task of defect detection on an LCD image within 60 ms.

## 1. Introduction

Over the past decade, TFT-LCD has become a very popular flat panel display in our daily life due to its advantages over the CRT monitor such as lower power consumption and smaller volume. With the increase of demand, every LCD manufacturer has made efforts to produce LCD panels of high quality, especially for larger-size LCD panels. Recently, an inspection/integration department has been introduced to set in the LCD manufacturers in order to ensure product quality. Yet the product yield still has space to improve because the task of defect inspection heavily relies on human observers in current practice: the inspection reliability depends on experience and physical conditions of engineers. Therefore, automatic optical inspection (AOI) has become a solution for real-time and robust defect inspection, and inspection method/scheme also plays a critical role in AOI in addition to the hardware design of AOI equipment.

Previously, much of the literatures have dealt with the so-called mura defect, e.g., [1–3]. The mura defect may be spot-type, line-type, or even region-type, and can only be observed after LCD panels are driven to a constant gray level. Hence, the task of mura defect inspection can only be executed in the cell process, the second process of TFT-LCD manufacturing. However, once a panel is found to have mura defects, the panel will be discarded if not repairable, resulting in a great increase in production costs. In practice, most mura defects are due to the defects that already occur in the former process, the array process. For example, if the surface of a panel is scratched by a deformed cassette or glass particles in the array process, the gate electrode of the panel is most likely to become an open circuit. If this is the case, a line-type mura defect (usually a white line) will be observed in cell process. Fortunately, the panels with defects can still be fixed by rework if the defects in array process are detected in real time. Thus, building a scheme that can robustly detect defects from the surface images of panels, which is also the focus of this paper, is still critical to every LCD manufacturer to date, which is also the focus of this paper.

**Figure 1:**
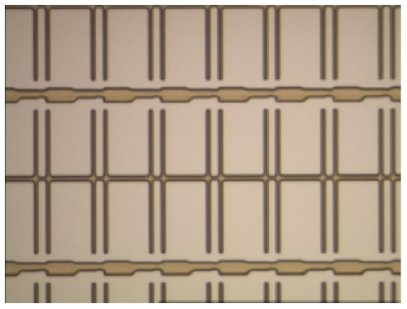
Image of a normal gate electrode (GE) pattern.

**Figure 2:**
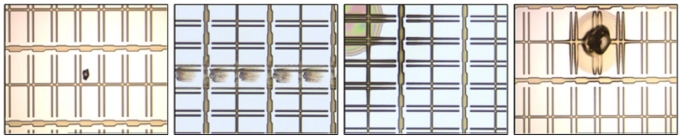
Examples of defect images.

**Figure 3:**
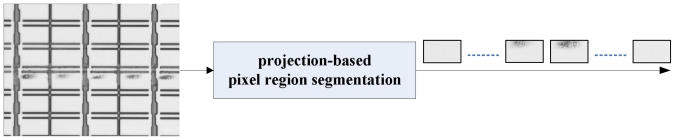
An illustrative example for pixel region segmentation.

The array process is the first process in TFT-LCD manufacturing which consists of five successive engineering processes: gate electrode (GE), semiconductor electrode (SE), source and drain (SD), contact hole (CH), and pixel electrode (PE) engineering. Each engineering process is responsible for generating a distinct pattern on a glass substrate. The image of a normal GE pattern is shown in Figure 1. Moreover, various defects would occur in each engineering process due to physical factors such as scratch, abnormal photo-resist coating, and particles. Figure 2 shows some defect images. There are many rectangle regions on an LCD panel (please refer to Figure 3 for clearer illustration). We call them pixel regions (PRs). The actual width of each PR is around 60 micrometers. If a defect appears in an image, the defect would appear within one single PR or simultaneously on several PRs in the image. Therefore, to judge whether the acquired image contains a defect or not, we just need to judge whether the PRs in the image are all normal. If all the PRs are found to be normal, the image is normal; otherwise the image is defective. Therefore, the defect detection problem can be regarded as a binary classification problem: normal PR and defective PR classification. When using a binary classifier to solve this problem, e.g., the support vector machine (SVM) [4], one has to collect a set of PRs for training. Normal PR patterns are easy to collect and they involve only small variations in uniformity. However, due to diverse defect modes and their occurrence frequencies, the available defective PRs are in general under-sampled. As a result, the true distribution of the defective PR patterns is difficult to obtain. Compared with binary classification strategy, one-class classification (also known as novelty detection) would be more appropriate when facing the situation where one of the two classes is under-sampled [5,6].

Liu *et al.* [7] have recently applied the one-class classification strategy to the defect detection in LCD array process, and achieved a high defect detection rate on the images in GE engineering. Their system is based on the locally linear embedding (LLE) [8] and the support vector data description (SVDD) [9], where LLE is used for dimensionality reduction and feature extraction, and SVDD serves as the defect detector. SVDD is a one-class machine learning method. It requires only normal PR patterns in its training stage. By introducing a kernel function satisfying the Mercer condition, SVDD is able to find a flexible boundary to tightly enclose all or most of the normal PRs in the original pattern space during training. Then, the boundary is used to distinguish normal PRs from defective PRs in the testing stage. If a test PR pattern falls inside of the boundary, it is accepted as a normal PR; otherwise it is rejected as a defective PR. While this SVDD-based decision making strategy is simple, it suffers from two critical problems related to testing time complexity and generalization performance.

1. *Testing time complexity.* The testing time complexity of SVDD is linear in the number of training patterns, which makes SVDD unable to classify a large number of test patterns within a short period of time, especially for the application of LCD array defect detection where the daily throughput is considerably high. A fast SVDD (F-SVDD) [10] has recently been proposed to address this issue.
2. *Generalization performance.* Recall that SVM embodies the principle of structural risk minimization in its formulation. Hence, SVM is capable of finding a hyperplane with maximum margin of separation in a kernel-induced feature space, thus having better generalization performance than the traditional learning machines based on empirical risk minimization. However, the formulation of SVDD does not consider the factor of class separation. More precisely, SVDD is unable to find a decision boundary with maximum margin of separation. The problem is not on the SVDD itself but due to the fact that only patterns of one single class are available during training in a one-class classification problem. Consequently, although SVDD can provide a target set with a compact description [11], satisfactory generalization performance cannot be guaranteed, which is a shortcoming of SVDD and remains to be solved.

Although SVDD has shown success in defect detection in [7], the detection rate still has space to improve. In addition, increasing product yield of 1% can save an LCD manufacturer at least one million US dollars per month, according to the internal evaluation of the LCD manufacturer we cooperate with. Accordingly, the issue of how to further improve generalization performance of SVDD would be worth studying from both the theoretical and practical aspects. In this paper, we present a method to address this issue by introducing a quasiconformal transform of a kernel and magnifying the Riemannian metric around the decision boundary of SVDD. The modified version is named quasiconformal kernel SVDD (QK-SVDD), which will be introduced in detail in Section 2. Then we apply the proposed QK-SVDD to the LCD array defect problem described above. Remarkable improvement in generalization performance has been indicated by our experimental results.

## 2. Results and Discussion

### 2.1. Basic Idea

According to real LCD manufacturing conditions, the number of normal LCD panels exceeds greatly the number of defective LCD panels. Therefore, the normal PRs greatly outnumber the defective PRs. As a result, the collected data set for training would be imbalanced if a two-class classification approach is adopted, the SVM by Vapnik [4] for example, the class imbalance problem occurs.

The class imbalance problem has attracted growing attention in the machine learning community. In a two-class classification problem, the class imbalance typically occurs when there are more instances of one (majority) class than the other (minority). This problem also occurs in a multi-class classification application if imbalances exist between the various classes. Most standard classification algorithms assume or expect balanced class distributions or equal misclassification costs. Consequently, those algorithms would tend to provide severely imbalanced degree of testing accuracy if the training set is severely imbalanced.

Previously, several workshops/special issues have been held/published to discuss and address this problem [12–15]. Various approaches for imbalanced learning have also been proposed, such as sampling (e.g., [16–18]), integration of sampling with ensemble learning (e.g., [19,20]), cost-sensitive learning (e.g., [21–23]), and SVM-based approach (e.g., [24–28]). These discrimination-based (two-class) approaches have shown to be useful in dealing with class imbalance problems. In addition, several works have also suggested that a one-class learning approach can provide a viable alternative to the discrimination-based approaches [29–33]. Interested readers can refer to [34] for a broad overview on the state-of-the-art methods in the field of imbalanced learning.

In practice, in addition to the class imbalance problem, the LCD defect detection also suffers from another critical problem resulting from the absence of negative information. To facilitate the following problem description, the normal PR class and the defective PR class are defined as the positive class and negative class, respectively.

**Figure 4:**
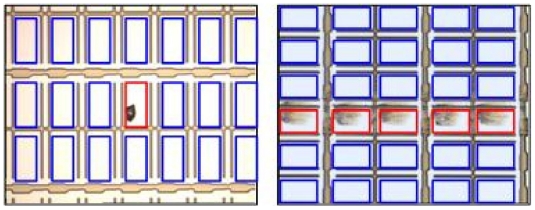
Different defect images contain different numbers of defective pixel regions (PRs). The normal and defective PRs are bounded with blue and red rectangles, respectively.

The main difference between a normal PR and a defective PR is that their appearances are apparently different, as can be observed from Figure 4. The color (or gray level) of a normal PR is nearly uniform, implying that the variation of the gray-level distribution of normal PRs is very small. On the contrary, the surfaces of defective PR not only contain various kinds of textures, but also vary greatly in color, implying that the variation of the true distribution for negative class in the data space is very large. Collecting a set of positive training data that can represent the true distribution of positive class is easy, because: (1) the variation of positive-class distribution is very small; and (2) most of the LCD panels are normal (the number of normal PRs is considerably large). Therefore, the positive class can be well-sampled during the data collection stage in real practice. However, representative defective PRs are difficult to obtain in practice for several reasons. For example, there are numerous types of defects in array process, more than 10 types at least. However, not all the defects would occur frequently. Some of the defects seldom appear, for example the defect caused by abnormal photo-resist coating (APRC). The defect “APRC” seldom occurs, because equipment/process engineers maintain the coating machines periodically. Even so, the coating machines might still break down occasionally. As a result, the number of available images containing the APRC defects is quite limited. But, the APRC defect has a large variation in color and texture. Unfortunately, limited APRC examples cannot stand for all kinds of APRC defects. Therefore, the collected negative training data are most likely under-sampled. Here, the “under-sampled” means that the collected negative training set cannot represent the true negative-class distribution in the data space, which is the problem of absence of negative information. Due to this problem, numerous false positive (*i.e.*, missing defects) will be produced if a two-class classification approach (e.g., a binary SVM) is applied to the LCD defect detection, which has been evidenced by the results reported in [7]. Compared with two-class classification approach, novelty detection approach is a better choice.

Novelty detection is one-class classification [10,35], which is to solve the conventional two-class classification problems where one of the two classes is under-sampled, or only the data of one single class can be available for training [5,6,9–11,35–40]. As analyzed above, for the LCD defect detection application, the normal PRs can be well-sampled, while the defective PRs are in general undersampled. Therefore, the LCD defect detection can be treated as a typical novelty detection problem. Accordingly, one-class classification is a better solution.

To summarize, it can be seen that the LCD defect detection suffers from two problems simultaneously: one is the class imbalance problem, and the other is the problem of the absence of negative information. For the first problem, there have been many sophisticated solutions, including sampling, cost-sensitive learning, SVM-based, and one-class learning approaches. However, the only solution to the second problem is the novelty detection approach (*i.e.*, one-class classification approach). Therefore, one-class classification would be a more appropriate approach to the LCD defect detection application.

One-class classifiers (also called novelty detectors) are to find a compact description for a class (usually being referred to *target class*). So, a one-class classifier is trained on the target class alone. In a testing stage, any points that do not belong to this description are considered as outliers. In this paper the normal PRs are treated as target data, while defective PRs are treated as outliers.

There are several approaches for one-class classification, such as density approach (e.g., Gaussian mixture model [5]), boundary approach (e.g., SVDD [9] and one-class SVM [40]), neural network approach [6,36], and reconstruction-based approach (e.g., the kernel principal component analysis for novelty detection [35]). It has been proven in [9] that when a Gaussian kernel is used, the SVDD proposed by Tax and Duin [9] is identical to the one-class SVM proposed by Schölkopf *et al.* [40]. This paper focuses on the SVDD since it has been applied to the same application in the works of [7] and [10], and has shown to be effective in detecting defective PRs. However, as discussed in Section 1, generalization performance of SVDD is limited. Therefore, the intent of this paper is on proposing a method to improve generalization performance of SVDD, and applying the improved SVDD to the LCD defect detection treated as a novelty detection problem. The improved SVDD is called quasiconformal kernel SVDD (QK-SVDD). Note that the QK-SVDD and SVDD are not two independent classifiers. To obtain QK-SVDD, one has to train an SVDD first, which will be introduced in Section 2.4. In the following part of the paper, we first introduce the defect detection scheme, and then derive the proposed method in details.

### 2.2. Overview of the Defect Detection Scheme

**Figure 5:**
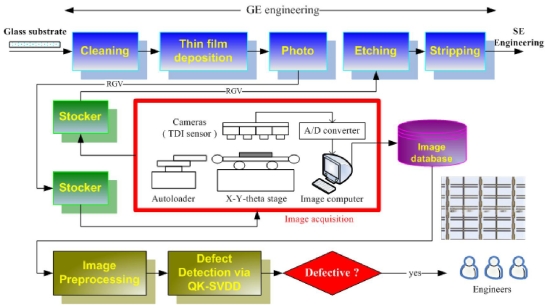
Overview of the defect detection scheme.

The array process consists of five engineering processes, each of which contains the same five processes, including cleaning, thin film deposition, photolithography (which contains three sub-processes: photo resist coating, exposure, and developing), etching, and stripping. By taking GE engineering as example, in the following we introduce our defect detection scheme depicted in Figure 5.

#### 2.2.1. Image Acquisition

After a sheet of glass substrate containing six LCD panels completes the photo process, it will be carried to a stocker by a rail-guided vehicle (RGV). At the stocker, a cassette containing 25 sheets of glass substrates is carried to the inspection equipment. After the cassette arrives, the inspection equipment will start to randomly pick six out of the 25 sheets, and each of the six chosen substrates will be put on an X-Y-theta stage by an autoloader, one at a time. Above the stage, there are four TDI (Timing Delay Integration) line-scan cameras equipped on the inspection equipment. The cameras begin to scan its surface once a sheet of glass substrate is placed on the stage. The scanned analog signals are transferred to digital signals (images) via an analog-to-digital (A/D) converter. Usually, it would take around 4 minutes to scan a sheet of glass substrate. These images will be stored in the image computer temporarily. After the six glass substrates are scanned, all the digital images will be stored in an image database. Each image is a 768 × 576 pixel 24-bit/pixel colored image (JPEG format), and has the resolution of around 1.15 (pixels/μm). Finally, the cassette will be carried back to the stocker, and sent to the next process, *i.e.*, the etching process. Note that the inspection equipment is placed between photo and etching processes because the defective panels can still be repaired by rework as long as they have not yet been sent into etching process.

#### 2.2.2. Image Preprocessing

Our scheme starts to access the images from the image databases, one image at a time. The colored image is first transformed into a gray-level one. Following that, the PRs are automatically segmented from the gray-level image by the projection-based PR segmentation method developed in [7]. The segmented PR images are then resized to have the same size of 30 × 30 pixels. Then, each PR image is represented by a vector (a datum) of 900 × 1 after row-by-row scanning. Finally, the PR data are sent into the QK-SVDD for further classification, one PR datum at a time.

#### 2.2.3. Defect Detection via QK-SVDD

Once the QK-SVDD receives a PR datum, it starts to judge whether the PR datum belongs to the class “normal”. If the PR datum is classified as the class “normal”, our scheme ignores this classification result; otherwise our scheme will output the result to engineers in the dust-free room via intranet because the PR is defective. The engineering can repair the defective PR in real time and diagnose the production machines to prevent the forthcoming LCD panels from suffering the same problem, thus being able to improve the yield rate significantly.

### 2.3. SVDD

In order to facilitate the following introduction, a normal PR datum is simply called a target datum, and a defective PR datum is called an outlier hereafter.

Given a target training set *T* = {**x***_i_* ε **R***^d^* }*_i=_*_1_ *^N^*, where **x***_i_* are target training data and *d* is the dimension of the space (*d* = 900), SVDD first maps the training data into a higher-dimensional feature space *F* from the input space *S* = **R***^d^* by a nonlinear mapping *φ*, and then finds a minimum-volume sphere in *F* such that all or most of the mapped target data are tightly enclosed by the sphere, which can be formulated as the constrained optimization problem:

(1) $$\begin{array}{l} Minimize\;\;\;R^{2}+C\sum_{i=1}^{N}\xi_{i}\\ subject\;to\;\;\;||\varphi (x_{i})-a_{F}|{|}^{2}\leq R^{2}+\xi_{i};\;\;\;\xi_{i}\geq 0,\;\;\;\forall i, \end{array}$$

where *C* ε[1/ *N*,1] is the penalty weight; **a***_F_* and *R* are the center and the radius of the sphere in *F*, respectively; and *ξ_i_* are slack variables representing training errors. The dual of (1) is

(2) $$\begin{array}{l} Maximize\;\;\;\sum_{i=1}^{N}\alpha_{i}K(x_{i},x_{i})-\sum_{i=1}^{N}\sum_{j=1}^{N}\alpha_{i}\alpha_{j}K(x_{i},x_{j})\\ subject\;to\;\;\;\sum_{i=1}^{N}\alpha_{i}=1;\;\;\;0\leq \alpha_{i}\leq C,\;\;\;\forall i, \end{array}$$

where *α_i_* are Lagrange multipliers; and *K* is the kernel function defined by *K*(**x**, **y**) =*φ* (**x**)*^T^* *φ* (**y**). We consider only the Gaussian kernel *K* (**x**, **y**) = exp(− ||**x** − **y||**^2^ /2σ ^2^ ) in this paper, where σ is the width of Gaussian and a user-defined kernel parameter. The training data for which 0 <*α_i_* ≤ *C* are called support vectors (SVs). The center **a***_F_* of the sphere is spanned by the mapped training data:

(3) $$a_{F}=\sum_{i=1}^{N}\alpha_{i}\varphi (x_{i}),$$

and the radius *R* of the sphere can be obtained by taking any **x***_k_* ε UBSVs, to calculate the distance between its image *φ* (**x***_k_* ) and **a***_F_*:

(4) $$\begin{array}{l} R^{2}=\;||\varphi (x_{k})-a_{F}|{|}^{2}\\ =K(x_{k},x_{k})-2\sum_{i=1}^{N}\alpha_{i}K(x_{i},x_{k})+\sum_{i=1}^{N}\sum_{j=1}^{N}\alpha_{i}\alpha_{j}K(x_{i},x_{j}). \end{array}$$

For a test datum **x**, its output can be computed by the decision function:

(5) $$\begin{array}{l} f(x)=R^{2}-\;||\varphi (x)-a_{F}|{|}^{2}\\ =R^{2}-K(x,x)+2\sum_{i=1}^{N}\alpha_{i}K(x,x_{i})-\sum_{i=1}^{N}\sum_{j=1}^{N}\alpha_{i}\alpha_{j}K(x_{i},x_{j}). \end{array}$$

If *f* (**x**) ≥ 0, **x** is accepted as a target (a normal PR); otherwise it is rejected as an outlier (a defective PR). We can see from equation (5) that the decision function is nonlinearly related to the input data. Therefore, although the decision boundary *f* (**x**) = 0 is the sphere boundary in the feature space *F*, it is actually flexible (non-spherical) in the original space *S*, and thus being able to fit any irregular-shaped target sets.

### 2.4. QK-SVDD

Looking back at equation (1), we can see that SVDD does not consider the factor of class separation in its formulation, but consider simply the volume of the sphere in *F* and the number of target training errors. Thus, the decision boundary *f* (**x**) = 0 would be too close to the target set to give satisfactory generalization performance. In this paper, we propose a method to improve generalization performance of SVDD, which is based on the kernel geometry in the kernel-induced feature space *F*.

When a Gaussian kernel is used, the associated mapping *φ* embeds the input space *S* into an infinite-dimensional feature space *F* as a Riemannian manifold, and the kernel induces a Riemannian metric in the input space *S* [41,42]:

(6) $$g_{ij}(x)={\left(\frac{\partial \varphi (x)}{\partial x_{i}}\right)}^{T}\left(\frac{\partial \varphi (x)}{\partial x_{j}}\right)={\frac{\partial^{2}K(x,x^{\prime })}{\partial x_{i}\partial x_{j}^{\prime }}|}_{x^{\prime }=x},$$

where *x_i_* stands for the *i*th element of the vector **x**, and *g_ij_* (**x**) is the Riemannian metric induced by a kernel at **x**. The Riemannian distance *ds* in *F* caused by a small vector *d***x** in *S* is given by

(7) $${ds}^{2}=\sum_{i}\sum_{j}g_{ij}(x){dx}_{i}{dx}_{j}$$

Thus, the volume form in a Riemannian space can be defined as

(8) $$dV=\sqrt{\text{det}\{G(x)\}}{dx}_{1}{dx}_{2}\cdots {dx}_{d},$$

where $\sqrt{\text{det}\{G(x)\}}$ is a magnification factor, and *G* (**x**) is the matrix with elements *g_ij_* (**x**). Equation (8) shows how a local volume in *S* is magnified or contracted in *F* under the mapping of *φ*. Furthermore, a quasiconformal transformation of the Riemannian metric is given by

(9) $${\tilde{g}}_{ij}(x)=\Omega (x)g_{ij}(x),$$

where Ω (**x**) is a scalar function of **x**. To realize this transformation, it is necessary to find a new mapping *φ̃*. In practice, it is difficult to achieve this because the mappings are usually unknown in kernel methods. However, if *φ̃* is defined as

(10) $$\tilde{\varphi }(x)=D(x)\varphi (x),$$

where *D* (**x**) is a positive real-valued quasiconformal function, then we obtain a quasiconformal transformation of the original kernel *K* by using a simple kernel trick:

(11) $$\tilde{K}(x,x^{\prime })=D(x)D(x^{\prime })K(x,x^{\prime }),$$

where *K̃* is called quasiconformal kernel. Finally, substituting (11) into (6) yields the new metric *g̃_ij_* (**x**) associated with *K̃*:

(12) $${\tilde{g}}_{ij}(x)=(\partial D(x)/\partial x_{i})(\partial D(x)/\partial x_{j})+D{\left(x\right)}^{2}g_{ij}(x)$$

Suppose that the goal is to magnify the local volume around the image of a particular data point **x** ε *S*, the first step is to choose a function *D* (**x**) in a way that it is the largest at the position of *φ* (**x**) and decays with the distance from *φ* (**x**). By doing so, new Riemannian metric *g̃_ij_* (**x**) becomes larger around **x** and smaller elsewhere, as can be seen from equation (12). As a result, the local volume around *φ* (**x**) is magnified, and magnifying the volume around *φ* (**x**) is equivalent to enlarging the spatial resolution in the vicinity of *φ* (**x**) in *F*.

Recently, the technique of the quasiconformal transformation of a kernel has been applied to improve generalization performance of existing methods, including SVM [43], nearest neighbor classifier [44], and kernel Fisher discriminant analysis (KFDA) [45]. In this paper we present a way of introducing this technique into SVDD. The idea is as follows.

If we hope to improve generalization performance of SVDD, we need to increase the separability of classes (target and outlier), which can be achieved by enlarging the spatial resolution around the boundary of the minimum-enclosing sphere in *F*. According to the technique of quasiconformal kernel mentioned above, the function *D* (**x**) should be chosen in a way that it is the largest at the sphere boundary and decays with the distance from the sphere boundary in *F*. However, the difficulty is that we do not know where the sphere boundary is located, because the feature space *F* is actually implicit. Nevertheless, there is an indirect way. According to the Kuhn-Tucker (KT) conditions

(13) $$\begin{array}{l} \alpha_{i}[R^{2}+\xi_{i}-{\left(\varphi (x_{i})-a_{F}\right)}^{T}(\varphi (x_{i})-a_{F})]=0,\forall i\\ \xi_{i}(C-\alpha_{i})=0,\forall i, \end{array}$$

the SVs can be divided into two categories: 1) the images of the SVs with 0 < *α_i_* < *C* are on the sphere boundary, and 2) the images of the SVs with *α_i_* = *C* fall outside the sphere boundary. The SVs in the first category called unbounded SVs (UBSVs), and the ones in the second category are called bounded SVs (BSVs). Since the mapped UBSVs lie exactly on the SVDD sphere boundary in *F*, increasing the Riemannian metric around the UBSVs in *S* is therefore equivalent to enlarging the spatial resolution in the vicinity of the sphere boundary in *F*. As a result, the separability of classes is increased, and generalization performance of SVDD is improved.

Accordingly, we can choose the function *D* (**x**) to have larger values at the positions of the mapped UBSVs and smaller elsewhere. Following the suggestion from [28], the quasiconformal function *D* (**x**) here is chosen as a set of Gaussian functions:

(14) $$D(x)=\sum_{x_{i}\in \text{UBSVs}}\text{exp}\left(-\frac{||\varphi (x)-\varphi (x_{i})|{|}^{2}}{\tau_{i}^{2}}\right),$$

where the parameter ***τ****_i_* is given by

(15) $$\tau_{i}^{2}=\frac{1}{M}\sum_{n}||\varphi (x_{n})-\varphi (x_{i})|{|}^{2}.$$

The parameter *τ_i_* ^2^ computes the mean squared distance from *φ* (**x***_i_* ) to its *M* nearest neighbors *φ* (**x***_n_* ), where **x***_n_* ε*UBSVs*. We set *M* = 3 in this study. As can be seen from (14), the function *D* (**x**) decreases exponentially with the distance to the images of the UBSVs.

In summary, the QK-SVDD consists of three training steps:

1. First, an SVDD is initially trained on a target training set by a primary kernel, thereby producing a set of UBSVs and BSVs. The primary kernel is the Gaussian kernel.
2. Second, the primary kernel is replaced by the quasiconformal kernel defined in equation (11).
3. Then, retrain the SVDD with the quasiconformal kernel using the same target training set.

After training the QK-SVDD, a set of new Lagrange multipliers, *α̃*_1_,···,*α̃_N_*, will be obtained. A new enclosing sphere with center ${\tilde{a}}_{F}=\sum_{i=1}^{N}{\tilde{\alpha }}_{i}\tilde{\varphi }(x_{i})$ and radius *R̃* will also be obtained. Finally, we arrive at the decision function of QK-SVDD:

(16) $$\begin{array}{l} \tilde{f}(x)={\tilde{R}}^{2}-||\tilde{\varphi }(x)-{\tilde{a}}_{F}|{|}^{2}\\ ={\tilde{R}}^{2}-\left(\tilde{\varphi }{\left(x\right)}^{T}\tilde{\varphi }(x)-2\sum_{i=1}^{N}{\tilde{\alpha }}_{i}\tilde{\varphi }{\left(x\right)}^{T}\tilde{\varphi }(x_{i})+\sum_{i=1}^{N}\sum_{j=1}^{N}{\tilde{\alpha }}_{i}{\tilde{\alpha }}_{j}\tilde{\varphi }{\left(x_{i}\right)}^{T}\tilde{\varphi }(x_{j})\right)\\ ={\tilde{R}}^{2}-\left(\tilde{K}(x,x)-2\sum_{i=1}^{N}{\tilde{\alpha }}_{i}\tilde{K}(x,x_{i})+\sum_{i=1}^{N}\sum_{j=1}^{N}{\tilde{\alpha }}_{i}{\tilde{\alpha }}_{j}\tilde{K}(x_{i},x_{j})\right)\\ ={\tilde{R}}^{2}-D{\left(x\right)}^{2}+2\sum_{i=1}^{N}{\tilde{\alpha }}_{i}D(x)D(x_{i})K(x,x_{i})-\sum_{i=1}^{N}\sum_{j=1}^{N}{\tilde{\alpha }}_{i}{\tilde{\alpha }}_{j}D(x_{i})D(x_{j})K(x_{i},x_{j}) \end{array}$$

Note that for the Gaussian kernel, *K*(**x**, **x**) = 1, ∀**x** ε **R***^d^*. For a test data point **x**, it is classified as a target if *f̃* (**x**) ≥0 ; an outlier otherwise. Also note that the last term is a constant. Therefore, the testing time complexity of QK-SVDD, similar to SVDD, is also linear in the number of training data.

### 2.5. Comparison between Our Method and the Kernel Boundary Alignment (KBA) Algorithm

Here we compare our method with the KBA algorithm proposed by Wu and Chang [28], since the KBA algorithm is also based on the quasiconformal transformation of a kernel.

Recall that when a binary SVM is trained on an imbalanced data set, the learned optimal separating hyperplane (OSH), denoted as *f* (**x**) = 0, would be skewed toward the minority class in a kernel-induced feature space. The KBA was designed to deal with the class-boundary-skew problem due to imbalanced training data sets. The KBA algorithm consists of two steps. In the first step, the KBA algorithm estimates an “ideal” separating hyperplane within the margin of separation by an interpolation procedure. The ideal hyperplane and the OSH are parallel to each other, but may be different in location. If the training data set is balanced, the estimated ideal hyperplane and the OSH will be the same; otherwise, compared with the OSH, the estimated (or interpolated) ideal hyperplane should be closer to the majority support-instance hyperplane, defined as *f* (**x**) = −1 in [28], such that the class-boundary-skew problem due to the imbalanced training data set can be solved. Assuming that the distance between the ideal hyperplane and the OSH is *η*, the objective of this step is to find the optimal value of *η* subject to the constraint: 0 ≤ *η* ≤ 1. Therefore, the interpolation procedure is formulated as constrained optimization problem (see [28] for details). Then, in the second step, the KBA algorithm chooses a feasible conformal function to enlarge the spatial resolution around the estimated ideal hyperplane in the feature space.

The advantages of the KBA-based SVM over the regular binary SVM is two-fold: not only the class-boundary-skew problem due to imbalanced training data sets can be solved, but also the generalization performance can be improved simultaneously.

The design of KBA is based on information of separation margin in the interpolation procedure. Without this information, this procedure cannot be formulated as a constrained optimization problem, and as a result, the location of the ideal hyperplane cannot be estimated. Therefore, the KBA algorithm cannot be applied to SVDD, since SVDD is trained on a single target class alone: there is no such margin of separation. The decision boundary learned from SVDD is simply a sphere boundary in the feature space.

The main difference between the KBA and our method is that the KBA is designed for binary classifier SVM while our method is designed for one-class classifier SVDD. The common is that both KBA and our method are based on the technique of quasiconformal transformation of a kernel. Although our method is much simpler, it works, as demonstrated in the next section.

## 3. Experimental Section

According to the introduction to the defect detection scheme in Section 2, we see that the performance of the scheme highly depends on the defect detector. Therefore, in this subsection, we conduct several experiments to test performance of the proposed QK-SVDD.

### Data

**Figure 6:**
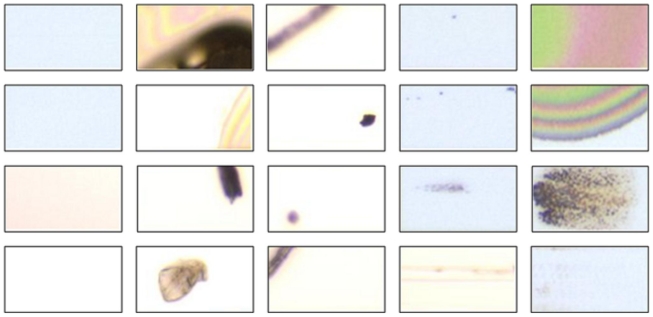
Examples of the chosen PRs in the experiment. The PRs in the first column are normal, while the rest are defective.

A total of 100 defect images are used in the experiment. They were captured in GE engineering in an array plant of a TFT-LCD manufacturer in Taiwan. There is a kind of defect in each image, and the defect occupies several PRs. The numbers of the PRs in different defect images may be different, and the numbers of the defective (or normal) PRs in different defect images may also be different. For example, the left image in Figure 4 contains 18 PRs in which one is defective and the remaining 17 PRs are normal. In the right image, there are 30 PRs in total, where the number of defective PRs and the number of normal PRs are 5 and 25, respectively. After performing PR segmentation on each image, we obtain 182 defective PRs and 1706 normal PRs in total. Examples of the normal and defective PRs are displayed in Figure 6. All the PR images are transformed into gray-level ones, and then resized to have the same size of 30 × 30 pixels. Finally, they are represented as vectors (data) of 900 dimensions.

### 3.1. Comparison based on Balanced Test Sets

Ten different runs are executed in the experiment. In each run, we randomly collect 200 data from the 1706 normal ones, and 100 data from the 182 defect ones. The collected 200 normal data and the 100 defect data form a target set and an outlier set, respectively. The first 100 data in the target set were used as target training data to train the methods to be compared. The remaining 100 target data and all the 100 outliers were used for testing the methods.

#### Training

In all the 10 runs, we set the penalty weight *C* to a constant (*C* = 0.4). Then, in each run we perform the following training procedure to determine the Gaussian kernel parameter ***σ***. The training strategy here follows the one suggested in [9]: ***σ*** is determined by decreasing its value from a large one (starting from a large ***σ*** is to ensure all the target training data are enclosed by the sphere at the very beginning) until a predefined target rejection rate *r* on the 100 target training data is reached. The larger the *r* is, the smaller the ***σ*** is. Defining such a threshold *r* ensures that a compact description for the target class can be obtained. However, *r* cannot be too large; otherwise the trained sphere will become too tight to get a good classification result. We set *r* to 0.01 and 0.05, respectively; for example, *r* = 0.05 means 5% of the target training data need to be rejected by the SVDD sphere in the training stage. Once the predefined threshold is reached, the training of SVDD is stopped, and the value of ***σ*** is fixed. Then, the same value of *σ* is used to train QK-SVDD. Clearly, the values of ***σ*** in the ten runs would not be the same because the target training sets in the runs are different.

#### Testing results

After training SVDD and QK-SVDD in each run, the prepared test set containing 100 target data and 100 outliers is then fed into the methods, and then three results for each of the two methods are obtained, including *target rejection rate* (TRR), *outlier acceptance rate* (OAR), and *error rate* (ER), defined as

$$\begin{array}{c} \text{TRR}=\frac{\#\text{target data that are rejected as outliers}}{\#\text{target data}}\\ \text{OAR}=\frac{\#\text{outliers that are accepted as targets}}{\#\text{outliers}}\\ \text{ER}=\frac{\#\text{target data that are relected as outliers}+\#\text{outliers that are accepted as targets}}{\#\text{target data}+\#\text{outliers}} \end{array}$$

After the ten runs are finished, the average results are obtained and listed in Tables 1 and 2. Note that the average ER is computed by (Average TRR + Average OAR)/2. According to the results, QK-SVDD performs better than SVDD in both cases (*r* = 0.01 and *r* = 0.05), especially in the case of *r* = 0.05 where QK-SVDD outperforms SVDD by 1.85% (5.85%–4.00%) in terms of average error rate. The improvement in average error rate reaches 31.62% (1.85/5.85), which demonstrates the validity of using QK-SVDD to improve generalization performance of the original SVDD.

In contrast with the average error rate, average outlier acceptance rate would be more important for engineers in practice. As aforementioned, an outlier represents a defective PR. If a defective PR is classified as a target (a normal PR), there will be no chance to repair the damaged LCD panel because the defective PR is not detected, thus increasing production cost. Hence, a good defect detector should be capable of achieving a low-enough outlier acceptance rate. We can observe from Table 2 that QK-SVDD achieves an average outlier acceptance rate of 3.60%, which is much lower than that of SVDD (6.10%). Moreover, improvement in average outlier acceptance rate is (6.10–3.60)/6.10 = 41.98%, which means that the production cost can be substantially reduced if the SVDD detector is replaced by the QK-SVDD.

#### Speed

During the experiment, the training time and testing time in each run are recorded in order to compare the speeds between SVDD and QK-SVDD. Table 3 lists the average training time and testing time. In our experiment, the methods are implemented with Matlab. A Pentium 2.80-GHz-CPU computer (with 4 GB RAM) running on Windows 7 is used.

Recall that QK-SVDD needs to train a SVDD by a Gaussian kernel and then retrain a SVDD by a quasiconformal kernel. Hence, it is easy to see that SVDD has only to solve the quadratic programming (QP) problem in equation (2) once, while QK-SVDD needs to solve the QP problem twice, which is the main reason that QK-SVDD has a higher training time complexity (1.468 s) than SVDD (0.623 s). The training time of QK-SVDD (1.468 s) is acceptable and can be actually ignored in the LCD inspection application because image acquisition takes much more time: inspection equipment takes around 4 minutes to scan a sheet of glass substrate.

However, the training time complexity of the QP problem is *O* (*N^3^*) 10]; hence, it is expected that QK-SVDD will be computationally expensive in training if the proposed QK-SVDD is applied to other problems where the training dataset is large-scale, e.g., the extended MIT face dataset [46]. A method to reduce training time complexity of QK-SVDD is required; however, it is beyond the scope of this work.

On the other hand, QK-SVDD spends only 2.38 ms accomplishing the task of classification on a PR datum. Also, an LCD image contains around 25 PRs in average. Therefore, QK-SVDD is able to accomplish the defect-detection task on an LCD image within 60 ms on average.

### 3.2. Comparison Based on Imbalanced Test Sets

In this subsection, we further compare the two methods on imbalanced test sets with ten different runs in the experiment. In each run, we randomly collect 1000 data from the 1706 normal ones, and 100 data from the 182 defect ones. The collected 1000 normal data and the 100 defect data form a target set and an outlier set, respectively. The first 100 data in the target set are used as target training data to train the methods to be compared. The remaining 900 target data and all the 100 outliers were used for testing. During the training stage, we set *r* to be 0.01, 0.05, and 0.1, respectively. In each run, a TRR and an OAR on the test set are obtained. However, since the test set in each run is highly imbalanced, hence, compared with the usual classification error rate adopted in the last experiment on balanced test sets, the balanced loss described in [47] would be a more appropriate performance measure for imbalanced test sets [10,48]. The balanced loss (BL) is defined as

(17) $$\begin{array}{l} \text{BL}=1-\frac{\text{TAR}+\text{ORR}}{2}\\ =\frac{\text{TRR}+\text{OAR}}{2}, \end{array}$$

where TAR and ORR denote the target acceptance rate and outlier rejection rate, respectively, and TAR = 1 − TRR and ORR = 1 − OAR. The average TRR and average ORR over the ten runs are listed in Table 4 (*r* = 0.01), Table 5 (*r* = 0.05), and Table 6 (*r* = 0.1). Note that the average BL is computed by (Average TRR + Average OAR)/2.

From Table 4 to Table 6, we can see that the QK-SVDD outperforms the original SVDD under the three different settings (*r* = 0.01, *r* = 0.05, and *r* = 0.1). It is worth noticing that altering r to a larger value does not necessarily lower the average balanced loss. For example, as *r* is increased from 0.05 to 0.1, the values of the average BL of SVDD and QK-SVDD decrease. The main reason is that the average TRR increases substantially and simultaneously. However, the BL (or classification error) is not the major concern for engineers. The most important performance index is the defect detection rate defined as:

Defect Detection Rate=1-OAR.

In order to maintain high competiveness in the market, quality is one of the most important factors. If most of the defective LCD panels can be found and repaired immediately, the product yield rate can thus be improved significantly. Therefore, For an LCD manufacture, the defect detection rate would be the most important. In current practice, the LCD manufacture has made a specification for the defect detection rate: it should be larger than 99%. Furthermore, under this condition, the false alarm rate should be as small as possible: a false alarm means a normal PR is classified as a defective PR. Namely, false alarm rate = TRR.

We can observe from Table 6 that when *r* = 0.1, the original SVDD obtains a low enough average OAR (2.20%): thus the average defect detection rate is 97.8%. However, the specification made by the manufacturer is still not satisfied. On the contrary, the QK-SVDD (99.2%) satisfies this specification. Also, compared with the original SVDD, the QK-SVDD achieves a lower false alarm rate (7.54%). Actually, if the value of *r* is increased further, say *r* = 0.2, it can be expected that the defect detection rate of SVDD or QK-SVDD will go to 100% (or a value near 100%). However, it is not necessary to do so, because the defect detection rate of QK-SVDD has been high enough (99.2%) as *r* = 0.1. More importantly, it can also be predicted that as *r* is increased further, say *r* = 0.2, the resulting false alarm rate will become too high. If it is too high, engineers will spend much time checking a great number of LCD panels that need not to be checked, which would rise the product cycle time substantially. Consequently, product throughput, which is also a key practical consideration in addition to product quality, will therefore be decreased.

## 4. Conclusions

In this paper we have presented a defect inspection scheme for TFT-LCD array process. The core of the scheme is a defect detector. A novel one-class classifier called quasiconformal kernel SVDD (QK-SVDD) has been proposed as the defect detector. The QK-SVDD is designed to overcome the weakness of the original SVDD in generalization performance. Experimental results carried out on real LCD images have indicated that the proposed QK-SVDD substantially improves generalization performance of SVDD in the LCD inspection application, and the improvement in generalization performance is considerably significant, over 30%. In addition, the QK-SVDD defect detector is able to obtain a low defect-detection error rate 4% on pixel region images, and classify each pixel region image within 3 ms.

In this paper, the pixel region images are directly fed into QK-SVDD for classification without any feature extractions. We believe that the error rate can be further improved by introducing useful feature extraction methods into the defect detection scheme, such as the discrete cosine transform (DCT) [3] and the kernel principal component analysis [49]. The feature evaluation task will be one of our future works.

## Figures and Tables

**Table 1:** Comparison of Testing Performance between support vector data description (SVDD) and quasiconformal kernel (QK)-SVDD (*r* = 0.05).

Methods	Average TRR (in %)	Average OAR (in %)	Average ER (in %)
SVDD	1.10 (±0.34)	12.80 (±2.74)	6.95
QK-SVDD	0.90 (±0.27)	10.70 (±1.95)	5.80

**Table 2:** Comparison of Testing Performance between SVDD and QK-SVDD (*r* = 0.01).

Methods	Average TRR (in %)	Average OAR (in %)	Average ER (in %)
SVDD	5.60 (±1.54)	6.10 (±2.14)	5.85
QK-SVDD	4.40 (±1.34)	3.60 (±1.74)	4.00

**Table 3:** Comparison of Training and Testing Time between SVDD and QK-SVDD.

	Average Training Time (s)	Average Testing Time (ms/PR)
SVDD	0.623	2.16
QK-SVDD	1.468	2.38

**Table 4:** Comparison of Performance on Imbalanced Test Sets (*r* = 0.01).

Methods	Average TRR (in %)	Average OAR (in %)	Average BL (in %)
SVDD	0.92 (±0.44)	11.60 (±2.71)	6.26
QK-SVDD	0.89 (±0.41)	10.10 (±1.69)	5.45

**Table 5:** Comparison of Performance on Imbalanced Test Sets (*r* = 0.05).

Methods	Average TRR (in %)	Average OAR (in %)	Average BL (in %)
SVDD	5.23 (±1.23)	5.58 (±1.87)	5.41
QK-SVDD	4.21 (±1.08)	3.70 (±1.77)	3.96

**Table 6:** Comparison of Performance on Imbalanced Test Sets (*r* = 0.1).

Methods	Average TRR (in %)	Average OAR (in %)	Average BL (in %)
SVDD	9.81 (±1.95)	2.20 (±1.01)	6.01
QK-SVDD	7.54 (±1.31)	0.80 (±0.43)	4.17
